# Retraction of ‘COUP-TF interacting protein 2 represses the initial phase of HIV-1 gene transcription in human microglial cells’

**DOI:** 10.1093/nar/gkad358

**Published:** 2023-05-09

*Nucleic Acids Research*, Volume 33, Issue 7, 1 April 2005, Pages 2318–2331, https://doi.org/10.1093/nar/gki529

The Editors were alerted in June 2022 about potential issues with Figures 3B, 5C and 6B as detailed below.

- Figure 3B: GST bands in the lower two panels appear to be identical.
- Figure 5C: There may be signs of splicing around the two COUP-TF bands.
- Figure 6B: Lanes 1 and 5 appear to be similar after horizontal flip.

The Editors began their investigation, during which the authors confirmed they no longer have the original, underlying data for any figure contained in the published article, as the research was conducted approximately 20 years ago. The authors also voluntarily disclosed that the upper panels of Figure 5B had ‘been built with at least 14 panels of different gels’.

An Expression of Concern was posted in November 2022. The Editors subsequently referred the matter to the institution where the research was conducted for investigation. The institution enlisted the help of independent external experts to scrutinize the figures objectively. They concluded:

1. The allegations are plausible and could involve the manipulation of several published images beyond accepted peer-reviewed methodological standards.
2. In the absence of the original gels, it is not possible to validate some of the conclusions of the article based solely on the experimental data produced in the article.
3. They are unable to lift all doubts about the conclusions of the article.

Based on their own assessment of the figures and the external experts’ reports, the Editors have lost confidence in the integrity of the data presentation in this paper and are ultimately retracting it.

